# Parental academic involvement in adolescence as predictor of mental health trajectories over the life course: a prospective population-based cohort study

**DOI:** 10.1186/s12889-015-1977-x

**Published:** 2015-07-14

**Authors:** Hugo Westerlund, Kristiina Rajaleid, Pekka Virtanen, Per E. Gustafsson, Tapio Nummi, Anne Hammarström

**Affiliations:** Stress Research Institute, Stockholm University, SE-106 91 Stockholm, Sweden; Department of Public Health and Clinical Medicine, Social Medicine, Umeå University, SE-901 85 Umeå, Sweden; Institute for Advanced Social Research, University of Tampere, FI-33014 Tampere, Finland; School of Information Sciences, University of Tampere, FI-33014 Tampere, Finland

**Keywords:** Mental health, Parental interest, Homework assistance, Academic achievement, Prospective, Longitudinal, Cohort study, Latent class trajectory modelling

## Abstract

**Background:**

Mental health problems are rising, especially among younger people, indicating a need to identify determinants of the development of mental health over the life course. Parental involvement in their children’s studies, particularly in terms of academic socialisation, has been shown to predict better mental health in adulthood, as well as other more favourable health outcomes, but no study published so far has examined its impact on trajectories of mental health. We therefore sought to elucidate the role of parental involvement at age 16 on the life course development of internalised mental health symptoms.

**Methods:**

In a population-based cohort (452 women and 488 men, 87 % of the eligible participants), we examined the association between parental involvement in their offspring’s studies, measured by teacher and pupil ratings at age 16, and an index of internalised mental health symptoms at the ages of 16, 18, 21, 30, and 43. Using latent class trajectory analysis, 5 different trajectories were derived from these indices: Very low stable (least symptoms), Low stable, Increasing, Moderate stable, and High decreasing (most symptoms). Multinomial logistic regression was used to regress trajectory membership on the parental involvement variables.

**Results:**

Teacher-rated parental interest in their offspring’s studies during the last year of compulsory school was associated with a lower risk of entering the Moderate stable (OR = 0.54; 95 % CI 0.30 to 0.98) and High decreasing (OR = 0.41; 0.18 to 0.91) trajectories, compared with the Low stable, also after adjustment for sex, parental social class and mental health, family unemployment and own school grades. Both these associations were present only in children with grades above the national average. Student-rated availability of assistance with homework was associated with a higher chance of entering the Very low stable trajectory in the whole sample (OR = 1.24; 1.07 to 1.43), in men (OR = 1.25; 1.05 to 1.48) and in those with above average grades (OR = 1.39; 1.13 to 1.72), and with a lower risk of entering the Moderate stable in women (OR = 0.74; 0.55 to 0.99), also after the same adjustments.

**Conclusions:**

Parental involvement in their offspring’s studies may buffer against poor mental health in adolescence which may track into adulthood.

**Electronic supplementary material:**

The online version of this article (doi:10.1186/s12889-015-1977-x) contains supplementary material, which is available to authorized users.

## Background

Mental health problems have become an increasingly salient cause of disability globally [1, 2]. There are indications that the increase in mental health problems has been particularly large in younger ages, especially among school age girls, at least in Western Europe [3, 4]. Mental health problems are known to track across the life course [5, 6]. It is therefore important to identify modifiable conditions in early life that can affect the long-term development of mental health.

In conjunction with the importance of academic performance, the role of the parents’ involvement in the schooling of their offspring, and its possible impact on academic success and mental health, has also been a topic under investigation. Academic achievement is known to be associated with mental health. School performance has been linked to a range of expressions of serious mental health problems later in life, including self-harm [7], completed and attempted suicide [7–10], drug dependence [11], eating disorder [12], and hospital admission due to major depression [13]. Fewer studies have examined the influence of early school performance on less severe but more frequent mental health problems in the population, and with seemingly less consistent associations, e.g., with depressive symptoms in young adulthood [14].

Parental involvement in the form of academic socialisation, such as parental interest, appears to incite the offspring’s own interest and motivation in the academic field and promote both academic achievement during school years [15, 16] and adult educational attainment [17], beyond other factors known to influence academic success or failure, such as childhood social class and cognitive ability [18]. Parental involvement has also been linked directly to mental health. A Greek study found that lack of parental interest in adolescent’s school/leisure activities was associated with both internalising and externalising mental health problems at age 18 [19]. Based on the 1958 British Child Development Study and the 1970 British Birth Cohort Study, Mensah and Hobcraft [20] found lack of parental interest in schooling at age 10–11 to be associated with both suboptimal self-rated health and poor mental well-being at the age of 30–33 years also after adjustment for a range of other exposures in childhood and adolescence.

A synthesis of nine meta-analyses indicated a positive effect of parental involvement on academic achievement regardless of how these concepts were defined, but concluded that the effect was strongest if the involvement entailed academic expectations, and weakest if it came in the form of homework assistance [17]. Some studies have found that assistance with homework may even be negatively affect achievement – possibly because it could be an attempt to compensate for pre-existing lower achievement, or because it may interfere with the child’s development of autonomy [15, 16].

Parental interest in their offspring’s schooling has been shown to be an independent predictor also of other aspects of adult health; teacher-rated parental interest in the child’s studies and parents reading to the child relate to better adult self-rated health in young adulthood [20, 21], and also to lower risk of obesity and indications of diabetes in middle-age [22].

In line with this, we have earlier found that teacher-rated parental interest in their offspring’s studies during the last year of compulsory school – rather than the parent’s social class or student-rated availability of practical academic support – predicted mid adulthood allostatic load, a measure of physiological wear and tear proposed to result from prolonged stress, in a cohort of Swedish school leavers [23]. Further adjustments indicated that academic achievement over the life course mediated a large part of the effect of parental interest on allostatic load. Mental health, which in contrast to allostatic load was measured repeatedly, is a possible life course link between parental interest and stress related physiological wear and tear, as well as a major health issue in itself.

We therefore sought to elucidate how parental involvement in their offspring’s studies at age 16 impacts on the development of internalised mental health symptoms from age 16 to 43. Our hypothesis was that academic socialisation, measured by teacher-rated parental interest in their offspring’s schooling, but not practical support, measured by student-rated availability of help with homework assignments, would predict more favourable trajectories of mental health. Since parental involvement may have a substantially different meaning and impact if it is focussed on compensating for poor school performance versus on encouraging already good performance, we tested our hypothesis also in an analysis stratified by mean school grades.

## Methods

### Sample

The sample was based on the Northern Swedish Cohort, a 27-year prospective study comprising all pupils in the ninth grade of compulsory school living in Luleå in 1981, when the participants where 16 years of age (*N* = 1083; 506 girls and 577 boys) [24]. The questionnaires were filled in during school hours, with the PI present and able to assist the pupils. Follow-up surveys were conducted in 1983, 1986, 1995 and 2008. Participants completed a comprehensive questionnaire at all follow-ups covering health, social and socioeconomic conditions, and school/working conditions. The majority of the items originated from the Swedish Survey of Living Conditions [25] and the Low-Income Study [26]. In order to allow follow-up, the questionnaires were not anonymised.

In 1981, personal interviews were conducted with all form teachers (*n* = 65) for each individual pupil by the project leader (AH). The interviewed teacher had been responsible for the class during the last three last years of compulsory school. The interviews were performed in a quiet room during school time. The teachers were interviewed with a previously used questionnaire consisting of 35 questions regarding the pupils' situation at school.

Of the original cohort, 1040 (96 %) subjects had enough information on mental health across the life course to be assigned to one of the trajectories described below, 951 (88 %) in addition had complete data on the studied exposures, and 940 (87 %) additionally had full information on all covariates, thus forming the analytic sample in the present paper.

### Measurements

**Social background** was assessed by the participants at age 16 using self-completion questionnaires. Social class was measured with two questions: *What is/was your father’s occupation?* and *What is/was your mother’s occupation?* Responses were coded according to the Swedish classification of social classes into working (blue-collar) class, lower white-collar class, and upper white-collar class. We defined parental social class as the highest of the father’s and the mother’s social class, with the highest value indicating upper class. Family unemployment was assessed by a *yes* response to the question *Has anyone in your family been unemployed during the last 12 months?* Parental mental health and alcohol problems were assessed with the question *Is your father healthy? (If he is dead: indicate how his health was most of the time when he was alive). Tick one or more options.* – and an identical question about the mother. The response options were: *Yes, as far as I know; No, (s)he has some kind of somatic disease; No, (s)he has psychological complaints; (S)He has alcohol problems; Don’t know.* Ticking psychological complaints and/or alcohol problems for father and/or mother was defined as parental mental health or alcohol problems.

**Parental interest in their offspring’s studies** was assessed with a question about *Knowledge about the parents’ interest in the pupil’s studies*, which was asked of the main teachers regarding each participant in 1981. There were five response options ranging from ‘probably very small’ to ‘probably very large’.

**Assistance with homework assignments** was based on the question *Can you get help with homework assignments when you need it?* with five response options from ‘no, never’ through ‘yes, always’ asked in the questionnaire to the participants themselves at age 16.

**Mean grades at exit from compulsory school** at age 16 were obtained from school records with the participants’ permission. Grades were given on a scale from 1 to 5 and by design normally distributed. The mean grades were thus also normally distributed and vary between 1 (the lowest possible) to 5 (the best possible) with 3.00 as the national mean. In some analyses mean grades were dichotomised as average or below vs. above average based on this theoretically defined national mean.

**Internalised mental health symptoms (IMHS) between the ages of 16 and 43 years**. In each of the five questionnaires the participants were asked if they had experienced any worry/anxiousness and anxiety/panic, in the past 12 months. The respondents were also asked how often, in the past 12 months, they had experienced sadness or feeling low. Based on these three questions, an ordinal scale index was created, ranging from 0 = not experiencing any symptoms to 8 = having worry/anxiousness, anxiety/panic and often or always feeling sad and low. A detailed description of the scale of IMHS has been published [27].

### Extraction of trajectories of mental health

We used latent class growth analysis (LCGA) to retrieve trajectories of IMHS from age 16 to 43. The analysis classifies individuals in the population into subgroups following distinct developmental trajectories of the outcome. Both the shapes and the number of trajectories are detected by the analysis. LCGA assumes that the variance and covariance for the latent growth factors within each class are zero, i.e., all individual within a class follow the same trajectory [28].

We considered growth trajectories with intercept, slope and a quadratic term, and tested models with two to six trajectory classes. Because a large proportion of individuals was clustered at the scale minimum each measurement time we assumed the distribution of the IMHS score to be censored normal [29]. The full information maximum likelihood method (FIML), assuming missing at random mechanisms, was used for handling missing data (default in the Mplus software). The trajectories were determined using information on IMHS only, but a sensitivity analysis adjusted for sex gave a similar solution with more than 99 % of the individuals assigned to the same latent class (data not shown).

We used several criteria to choose the optimal LCGA model for further analysis. As the Bayesian Information Criterion (BIC) [30] and the Lo-Mendell-Rubin (LMR) test [31] may favour less parsimonious models that are more difficult to interpret [29], we additionally considered entropy (value near 1 for a good model), size of the resulting classes (enough individuals in each to be meaningful in further analyses) and high posterior probabilities (near 1, on average, for a good model) and the interpretability of the fitted model [28]. In order to avoid local solutions that do not represent the global maximum of the likelihood, the analyses were repeated with increased number of random sets and start iterations, once plausible models were identified [28].

We considered the five class model to be the most sensible representation of the data. Compared with the three and four class models it demonstrated clearer heterogeneity and at least as high entropy (Additional file 1: Table S1). Adding a sixth class did not yield trajectories that were substantially different from those identified with the simpler models (Additional file 1: Figure S1). Although the BIC continued to increase when adding a sixth class (Additional file 1: Table S1), the increase was now smaller and the LMR test was only borderline significant. Moreover, the smallest class with this model was only 1.6 % (17 individuals). Altogether, we considered the five class model as optimal.

### Statistical analysis

Correlation coefficients were calculated for all pairs of variables in the study. To examine if parental involvement was related to the chance of following a certain trajectory of internalised mental health, we used multinomial regression to obtain odds ratios and 95 % confidence intervals for being assigned to the most likely trajectory as derived by LCGA. We analysed the whole sample as well as women and men separately, with parental social class (and in the former case also sex) as covariates. The individuals were weighted with the posterior probability of belonging to the assigned trajectory because ordinary statistical inference is not directly applicable for trajectory groups that are based on probabilities and thus are not fixed constructs. To test if parental involvement influences mental health independently of academic achievement, we entered mean school grades on leaving compulsory school in a final step of the analysis. The trajectories were derived using MPlus version 7.11; whereas all other analyses were done in IBM SPSS for Windows, version 20.0.0. All *p*-values are based on two-sided tests.

### Ethical approval

This study was conducted with the approval of the Regional Ethical Review Board in Umeå and has conformed to the principles embodied in the Declaration of Helsinki.

## Results

There were 940 participants in the analytic sample (87 % of the original sample), with 52 % men, and with 35 % belonging to the working class, 55 % middle class, and 10 % upper class (Table 1). 78 % had parents whose interest in their offspring’s education was rated as large or very large, with no sex difference, and 57 % had often or always been able to get help with their homework assignment; again with no sex difference. Girls had significantly higher school grades than boys, whose average closely matched the national average. Women had significantly higher ratings of internalised mental health symptoms at all ages and were less likely to belong to the Very low stable trajectory group. However, within both sexes, higher grades were associated with lower mental health symptoms (Table 2).

**Table 1:** Descriptive statistics for the sample

	Total	Women	Men	Test of sex difference
Total N (row %)	940	452 (48 %)	488 (52 %)	
Parental social class N (col %)				*p* = 0.332
Upper class	97 (10 %)	44 (10 %)	53 (11 %)	
Middle class	513 (55 %)	258 (57 %)	255 (52 %)	
Working class	330 (35 %)	150 (33 %)	180 (37 %)	
Unemployment in the family, N (%)	173 (18 %)	78 (17 %)	95 (20 %)	*p* = 0.400
Parental mental health or alcohol problems, N (%)	70 (7 %)	40 (9 %)	30 (6 %)	*p* = 0.136
Parental interest in offspring’s studies, N (col %)				*p* = 0.940
Probably very large	395 (42 %)	186 (41 %)	209 (43 %)	
Probably large	341 (36 %)	169 (37 %)	172 (35 %)	
Probably neither particularly large nor particularly small	144 (15 %)	69 (15 %)	75 (15 %)	
Probably small	43 (5 %)	21 (5 %)	22 (4 %)	
Probably very small	17 (2 %)	7 (2 %)	10 (2 %)	
Help with homework assignment possible, N (col %)				*p* = 0.115
Yes, always	282 (30 %)	144 (32 %)	138 (28 %)	
Yes, often	256 (27 %)	118 (26 %)	138 (28 %)	
Sometimes	271 (29 %)	134 (30 %)	137 (28 %)	
No, seldom	80 (8 %)	40 (9 %)	40 (8 %)	
No, never	51 (5 %)	16 (4 %)	35 (7 %)	
Mean grades on leaving compulsory school, mean (SD)	3.10 (0.75)	3.26 (0.73)	2.96 (0.76)	*p* < 0.0001
Average or below (≤3.0), N (col %)	407 (43 %)	170 (38 %)	237 (49 %)	*p* < 0.0001
Above average (>3.0)	533 (57 %)	282 (62 %)	251 (51 %)	
Latent trajectory of internalised mental health, N (col %)				*p* < 0.0001
Very low stable	254 (27 %)	61 (14 %)	193 (40 %)	
Low stable	525 (56 %)	275 (61 %)	250 (51 %)	
Increasing	77 (8 %)	51 (11 %)	26 (5 %)	
Moderate stable	54 (6 %)	41 (9 %)	13 (3 %)	
High decreasing	30 (3 %)	24 (5 %)	6 (1 %)	
Internalised mental health symptoms, mean (SD)				
At age 16	1.14 (1.39)	1.53 (1.62)	0.77 (0.99)	*p* < 0.0001
At age 18	1.44 (1.84)	2.03 (2.06)	0.89 (1.40)	*p* < 0.0001
At age 21	1.22 (1.71)	1.50 (1.89)	0.96 (1.47)	*p* < 0.0001
At age 30	1.47 (2.13)	1.82 (2.33)	1.15 (1.88)	*p* < 0.0001
At age 43	1.52 (2.30)	1.90 (2.53)	1.14 (2.00)	*p* < 0.0001

**Table 2:** Bivariate Pearson correlations between all variables in the studies; women above the diagonal and men below

	SC	UE	MH	PI	HA	GR	16	18	21	30	43
Parental social class (SC)	1	−0.11^a^	−0.12^b^	0.28^b^	0.14^b^	0.19^b^	0.05	0.01	0.02	−0.00	0.01
Unemployment in family (UE)	−0.04	1	0.15^b^	−0.24^b^	−0.04	−0.12^a^	0.08	0.10^a^	0.09	0.04	0.05
Parental mental health or alcohol problems (MH)	−0.12^b^	0.05	1	−0.14^b^	−0.05	−0.10^a^	0.08	0.09	0.06	0.06	0.10^a^
Parental interest in offspring’s studies (PI) according to teacher	0.21^b^	−0.15^a^	−0.14^b^	1	0.04	0.45^b^	−0.10^a^	0.01	−0.05	0.03	−0.03
Help with homework assignments (HA) according to student	0.18^b^	−0.05	−0.04	0.05	1	−0.02	−0.12^a^	−0.04	0.01	−0.13^b^	−0.04
Mean grade on leaving compulsory school at 16 (GR)	0.25^b^	−0.11^a^	−0.14^b^	0.42^b^	0.08	1	−0.14^b^	−0.02	−0.02	−0.10^a^	−0.07
Internalised mental symptoms at 16 (16)	−0.02	0.16^b^	0.14^b^	−0.10^a^	−0.08	−0.12^b^	1	0.30^b^	0.34^b^	0.26^b^	0.17^b^
Internalised mental symptoms at 18 (18)	0.10^a^	0.06	0.03	0.04	−0.04	−0.03	0.32^b^	1	0.36^b^	0.25^b^	0.16^b^
Internalised mental symptoms at 21 (21)	0.06	0.13^b^	−0.03	−0.08	−0.01	−0.15	0.24^b^	0.35^b^	1	0.41^b^	0.34^b^
Internalised mental symptoms at 30 (30)	0.02	0.08	−0.00	−0.04	0.01	−0.03	0.18^b^	0.27^b^	0.27^b^	1	0.39^b^
Internalised mental symptoms at 43 (43)	−0.05	0/09	0.03	−0.12^b^	−0.05	−0.13^b^	0.18^b^	0.23^b^	0.26^b^	0.39^b^	1

Note: ^a^ *p* < 0.05; ^b^ *p* < 0.01

**Fig 1.**
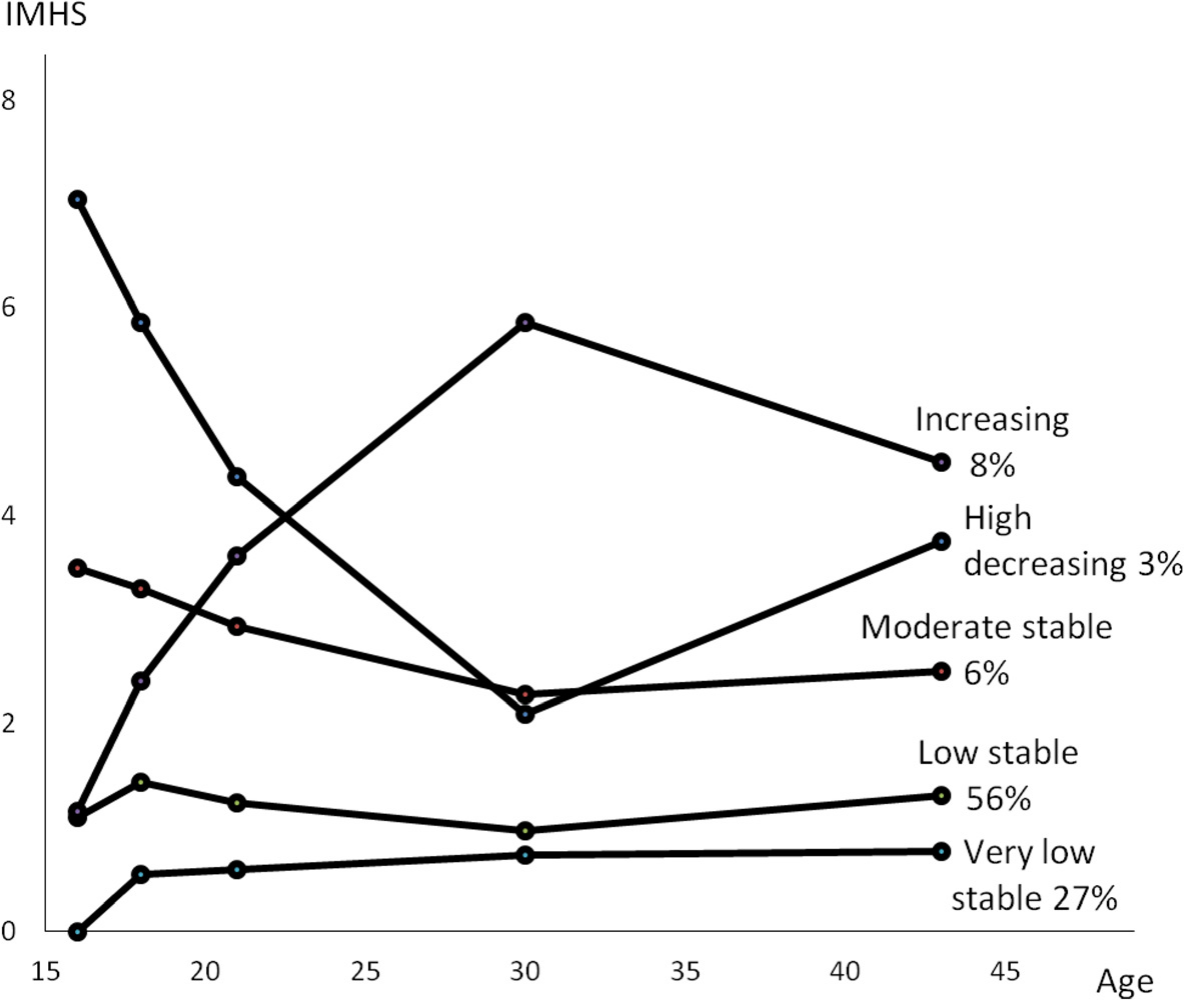
Latent trajectories of mental health symptoms from the age of 16 to the age of 43

Figure 1 portrays the shapes of the five trajectories obtained from the LCGA together with class sizes. The lowest trajectory class which we named ‘Very low stable’ included 27 % of the analytic sample. Individuals belonging to this class showed very low levels of internalised mental health symptoms (IMHS) throughout the follow-up. The ‘Low stable’ class showed slightly higher levels of IMHS and included 56 % of the sample. The ‘Moderate stable’ included 6 % and followed a trajectory with values of IMHS around 3. Finally, there were two classes that showed considerable changes in the IMHS between the ages 16 and 43: The ‘Increasing’ (8 %) started the same level as the Low stable class but progressed to high levels by age 30, decreasing somewhat by age 43. The smallest class, ‘High decreasing’ (3 %) showed very high levels of IMHS in the beginning, and lower, but still rather high levels later during follow-up.

Table 2 indicates a relatively strong association between teacher-rated parental interest in their offspring’s studies and grades (r = 0.45 in women, 0.42 in men, *p* < .0.01) but no association between student-rated help with homework assignment and grades. There was, however, virtually no association between parental interest and help with homework (r = 0.04–0.05, NS). In both women and men parental social class was moderately associated with parental interest, help with homework and grades (r = 0.14–0.28, *p* < 0.01), but not with IMHS except at age 18 for men (r = 0.10, *p* < 0.05). Parental interest, help with homework and mean grades were relatively weakly negatively correlated with IMHS at some ages. There were moderate correlations between IMHS at different ages, indicating some tracking.

In the whole sample, multinomial logistic regression (Table 3, upper panel) showed a significantly higher chance of entering the Very low stable trajectory (compared to the largest, Low stable trajectory) among those students who stated that they were able to get assistance with their homework assignments at age 16, an association that remained after adjustment for sex, parental social class and mental health problems, family unemployment, and own average school grades (OR = 1.24, 95 % CI 1.07 to 1.43, for each additional level of the exposure, range 0–4). Teacher-rated parental interest in their offspring’s studies, on the other hand, was associated with a lower risk of entering the High decreasing (worst) trajectory, an association which remained after adjustment for sex and parental social class and mental health problems, and family unemployment (OR = 0.65; 0.44 to 0.94), but was rendered non-significant by adjustment for grades.

**Table 3:** Parental interest in offspring’s studies and help with homework assignments at age 16 in relation to trajectories of internalised mental health symptoms from age 16 to 43 in women and men. Odds ratios (OR) with 95 % confidence intervals (CI) for belonging to the different trajectory groups derived from multinomial regression analyses with the two predictors mutually adjusted. The odds ratios denote the change in odds for each additional level of the exposure (range 0–4)

	Latent trajectory of internalised mental health symptoms OR (95 % CI)				
	Very low stable	Low stable	Increasing	Moderate stable	High decreasing
Whole sample					
***Parents’ interest*** *in their offspring’s studies at age 16 according to teacher*					
Model 1	1.02 (0.87 to 1.21)	Reference	1.00 (0.76 to 1.31)	0.77 (0.58 to 1.01)	**0.67 (0.47 to 0.94)**
Model 2	0.97 (0.82 to 1.16)	Reference	1.02 (0.76 to 1.36)	0.79 (0.59 to 1.06)	**0.65 (0.44 to 0.94)**
Model 3	0.93 (0.76 to 1.14)	Reference	1.15 (0.83 to 1.59)	0.88 (0.63 to 1.23)	0.89 (0.58 to 1.36)
***Help with homework*** *assignments at age 16 according to the student*					
Model 1	**1.23 (1.07 to 1.42)**	Reference	0.92 (0.74 to 1.14)	0.81 (0.64 to 1.03)	0.89 (0.64 to 1.22)
Model 2	**1.24 (1.07 to 1.43)**	Reference	0.91 (0.73 to 1.13)	0.81 (0.64 to 1.04)	0.86 (0.63 to 1.19)
Model 3	**1.24 (1.07 to 1.43)**	Reference	0.90 (0.73 to 1.13)	0.81 (0.64 to 1.04)	0.86 (0.62 to 1.18)
Adjusted R Square (Nagelkerke): Model 1: 0.146, Model 2:0.179 Model 3: 0.194					
Women					
***Parents’ interest*** *in their offspring’s studies at age 16 according to teacher*					
Model 1	1.04 (0.76 to 1.43)	Reference	1.04 (0.73 to 1.47)	**0.69 (0.50 to 0.95)**	0.78 (0.52 to 1.18)
Model 2	0.99 (0.71 to 1.39)	Reference	1.06 (0.73 to 1.54)	**0.67 (0.48 to 0.95)**	0.75 (0.47 to 1.18)
Model 3	0.92 (0.62 to 1.34)	Reference	1.26 (0.83 to 1.91)	0.72 (0.48 to 1.06)	1.01 (0.60 to 1.68)
***Help with homework*** *assignments at age 16 according to the student*					
Model 1	1.29 (0.98 to 1.70)	Reference	0.88 (0.66 to 1.16)	**0.75 (0.56 to 1.00)**	0.78 (0.54 to 1.13)
Model 2	1.27 (0.96 to 1.69)	Reference	0.86 (0.65 to 1.15)	**0.74 (0.56 to 0.99)**	0.76 (0.52 to 1.10)
Model 3	1.28 (0.97 to 1.71)	Reference	0.85 (0.64 to 1.13)	**0.74 (0.55 to 0.99)**	0.74 (0.52 to 1.08)
Adjusted R Square (Nagelkerke): Model 1: 0.044, Model 2: 0.074, Model 3: 0.101					
Men					
***Parents’ interest*** *in their offspring’s studies at age 16 according to teacher*					
Model 1	1.01 (0.83 to 1.24)	Reference	0.94 (0.61 to 1.45)	1.13 (0.60 to 2.10)	**0.43 (0.23 to 0.81)**
Model 2	0.96 (0.78 to 1.19)	Reference	0.94 (0.60 to 1.49)	1.28 (0.66 to 2.48)	**0.43 (0.22 to 0.87)**
Model 3	0.94 (0.74 to 1.19)	Reference	0.94 (0.55 to 1.60)	1.61 (0.79 to 3.28)	0.69 (0.29 to 1.64)
***Help with homework*** *assignments at age 16 according to the student*					
Model 1	**1.23 (1.04 to 1.45)**	Reference	0.98 (0.69 to 1.39)	0.97 (0.61 to 1.53)	1.27 (0.63 to 2.57)
Model 2	**1.25 (1.05 to 1.48)**	Reference	0.98 (0.69 to 1.40)	1.01 (0.64 to 1.61)	1.26 (0.62 to 2.55)
Model 3	**1.25 (1.05 to 1.48)**	Reference	0.98 (0.68 to 1.40)	1.01 (0.65 to 1.59)	1.28 (0.64 to 2.57)
Adjusted R Square (Nagelkerke): Model 1: 0.033, Model 2b: 0.093, Model 3: 0.105					
Lower than national average mean grades					
***Parents’ interest*** *in their offspring’s studies at age 16 according to teacher*					
Model 1	0.90 (0.72 to 1.13)	Reference	1.00 (0.69 to 1.43)	1.07 (0.74 to 1.56)	1.01 (0.64 to 1.59)
Model 2	0.85 (0.67 to 1.07)	Reference	0.96 (0.66 to 0.41)	1.07 (0.72 to 1.58)	0.93 (0.57 to 1.51)
Model 3	0.82 (0.63 to 1.05)	Reference	1.04 (0.71 to 1.54)	1.05 (0.70 to 1.58)	1.10 (0.67 to 1.82)
***Help with homework*** *assignments at age 16 according to the student*					
Model 1	1.09 (0.89 to 1.33)	Reference	0.77 (0.57 to 1.05)	0.78 (0.57 to 1.07)	0.84 (0.57 to 1.24)
Model 2	1.12 (0.91 to 1.38)	Reference	0.76 (0.56 to 1.03)	0.79 (0.57 to 1.08)	0.81 (0.55 to 1.20)
Model 3	1.12 (0.91 to 1.38)	Reference	0.76 (0.56 to 1.04)	0.79 (0.57 to 1.08)	0.83 (0.55 to 1.24)
Adjusted R Square (Nagelkerke): Model 1: 0.148, Model 2:0.209, Model 3: 0.237					
Higher than national average mean grades					
***Parents’ interest*** *in their offspring’s studies at age 16 according to teacher*					
Model 1	1.27 (0.90 to 1.78)	Reference	1.44 (0.80 to 2.59)	**0.54 (0.32 to 0.92)**	**0.41 (0.20 to 0.82)**
Model 2	1.26 (0.89 to 1.78)	Reference	1.58 (0.86 to 2.89)	**0.54 (0.30 to 0.95)**	**0.44 (0.20 to 0.97)**
Model 3	1.25 (0.87 to 1.78)	Reference	1.59 (0.85 to 2.98)	**0.54 (0.30 to 0.98)**	**0.41 (0.18 to 0.91)**
***Help with homework*** *assignments at age 16 according to the student*					
Model 1	**1.40 (1.14 to 1.72)**	Reference	1.08 (0.79 to 1.49)	0.81 (0.55 to 1.18)	0.90 (0.52 to 1.57)
Model 2	**1.39 (1.13 to 1.71)**	Reference	1.10 (0.79 to 1.52)	0.82 (0.56 to 1.22)	0.97 (0.53 to 1.76)
Model 3	**1.39 (1.13 to 1.72)**	Reference	1.10 (0.79 to 1.52)	0.82 (0.56 to 1.22)	0.97 (0.53 to 1.77)
Adjusted R Square (Nagelkerke): Model 1: 0.187, Model 2: 0.223, Model 3: 0.224					

Model 1: Adjusted for sex except in the sex stratified analysis

Model 2: Model 1 + adjustment for parental social class, family unemployment and parental mental health or alcohol problems

Model 3: Model 2 + adjustment for own average school grades at age 16

Note: Statistically significant results are marked in bold

Stratification by sex (Table 3, second and third panels) showed that men had a pattern very similar to that of the whole sample, with indications of a slightly stronger protective effect of parental interest against entering the High decreasing trajectory (OR = 0.43; 0.22 to 0.87), which, however, was also rendered insignificant after adjustment for grades. In women, both homework help and parental interest were associated with a lower risk of entering the Moderate stable (second or third worst) trajectory; the former association remained after full adjustment (OR = 0.74; 0.55 to 0.99) whereas the latter remained after adjustment for parental social class and mental health problems, and family unemployment (OR = 0.67; 0.48 to 0.95), but was rendered non-significant after adjustment for grades. Otherwise, the risk estimates were similar to those of men, but non-significant.

Stratification by mean grades (Table 3, fourth and fifth panels) revealed that parental interest and help with homework were associated with more favourable outcomes among those pupils who had above national average school grades, whereas all associations were non-significant for those with average or lower grades. In those with higher than average grades, teacher-rated parental interest was associated with a lower risk for entering both the Moderate stable (OR = 0.54; 0.30 to 0.98) and the High decreasing (OR = 0.41; 0.18 to 0.91) trajectories, and these associations remained after adjustment for both parental social class and own school grades (entered as a linear term). Student-rated help with homework assignments was associated with a higher chance of entering the Very low stable trajectory, an association which also remained in the fully adjusted model (OR = 1.39; 1.13–1.72).

## Discussion

In this prospective, community based cohort study we found that both teacher-rated parental interest in their offspring’s studies and student-rated availability of practical academic support during the last year of compulsory school (age 16) predicted more favourable trajectories of internalised mental health symptoms (IMHS) from the age of 16 to 43 years. These associations were mainly present among those who had above national average mean grades when they left compulsory school.

Parental involvement in their offspring’s schooling has previously been a topic of interest mostly within educational and developmental psychology, as a determinant of academic achievement in childhood and adolescence [15, 16], and a few studies have examined prospective associations also with adult educational attainment [18] or of adult health [20–23, 32]. Our findings extend these observations by illustrating how parental interest is reflected in the subsequent life course development of IMHS. While novel by utilising latent class trajectory modelling, this observation was according to our hypothesis that parental interest would have a positive effect on mental health, and agrees with earlier observations that lack of parental interest during teenage years is associated with poorer mental health in adulthood [19, 20].

Availability of help with homework assignments has previously not been consistently associated with improved outcomes later in life, as shown in our previous study in the same cohort [23], and as suggested by the literature, practical involvement of the parents is not necessarily a part of academic socialisation but can also negatively influence the child’s autonomy and academic development [15, 16]. However, in the present study, student-rated availability of help with homework was associated with a higher chance of entering the most favourable trajectory of IMHS. This was thus a somewhat surprising finding. Given that there is virtually no association between such help and school grades, it is unlikely that homework assistance affects mental health via academic achievement. However, help with homework could possibly reflect a positive parent–child relationships and a supportive family climate more broadly, and since help with homework assignments could also be offered by others than the parents (such as peers, teachers and older relatives) it is possible that it is also indicative of a generally supportive social network, which may enhance mental health among those who are already doing relatively well, but not necessarily buffer against more severe health problems.

The virtually non-existent correlation between teacher-rated parental interest and student-rated help with homework might seem counterintuitive, but although it is impossible to know exactly what the teachers and students were thinking of when answering the respective questions, it could be due to several factors influencing the rating of homework help in different directions: On the one hand, parents who are interested in their offspring’s studies should be more motivated to give practical help if needed compared with parents who are less interested, and it may also be that those who are interested are also more able as they may have pursued a more academic course in their own lives. On the other hand parental help might be triggered by academic problems that the student has, and thus actually be negatively correlated with academic achievement in some cases. Any association between parental interest and help with homework might be further diluted by the above-mentioned fact that homework help can also be given by others than the parents.

Another somewhat surprising finding was that both parental interest and availability of help with homework was significantly related to more favourable trajectories only in those participants who left compulsory school with above national average mean school grades. There are several possible explanations for this. In line with the reasoning in the preceding paragraph, it is possible that both homework help and parental interest in relation to less academically accomplished children is triggered by worry about the child’s development. If this is the case, then there could in fact be positive effects of such parental academic involvement which are masked by an association with other factors associated with negative mental health development. A second explanation could be that a high level of parental focus on academic achievement could be stressful for children with less developed academic abilities and possibly contribute to poor mental health, for instance by fuelling a sense of failure and inadequacy. On the other hand, it is also possible that children with more developed academic abilities are more able to utilise the support that they get from their parents, or that *lack* of support can be detrimental for the health of academically gifted children.

Women and men have very different levels of IMHS, but the patterns of association between parental interest and the trajectory classes are similar in the sexes, however with an apparently stronger protective effect against the Moderate stable trajectory in women and the High decreasing in men. Given the shape of these trajectories, this could possibly indicate a more long-lasting effect of parental interest in women compared to men.

A major strength of this prospective study is that it is based on a stable, age homogeneous cohort with very low attrition over the 27 years of follow-up. The cohort is population-based and has in various comparisons been found to be representative of this age cohort of the Swedish population [24]. Another strength is that we have employed latent class trajectory modelling to summarise the development of IMHS over a substantial part of the life course in such a way that patterns of heterogeneity can be revealed. A further strength is that two different types of data are used, decreasing the risk of common method variance: Parental interest in their offspring’s studies was based on an interview with the main teacher at age 16, whereas availability of home assignment help in school as well as IMHS were self-reported.

Our study also has some limitations. Using teacher evaluations of parental interest in their offspring’s studies has the advantage of avoiding some of the social desirability effect that could have been expected if the question had been asked of the parents or the students, and has the further benefit of utilising the teachers’ ability to compare parental interest between pupils. However, despite regular scheduled contacts between teachers and parents, teachers may not know exactly how interested the parents actually are, and their ratings could thus be confounded by for instance the student’s social background, socially conditioned behaviour, academic achievements or actual mental health problems. The high initial levels of mental health problems in the two trajectories most strongly predicted by teacher-rated parental interest could indicate that this is the case, but it would also be consistent with the interpretation that parental interest has a substantial but mostly short-term effect on mental health. In addition, we cannot rule out that the observed associations could partly be due to third variables rather than be causal. It is for instance possible that more mentally stable children, who are likely to continue on a more favourable trajectory, also motivate their parents to become more interested in their offspring’s studies.

Another weakness it that it is difficult to know if factors associated with mental health outcomes are confounders, mediators or underlying factors. This is especially the case with school grades, which indicate academic success, and is a likely mediator between parental involvement and mental health development. Adjustment for school grades could thus constitute over-adjustment which masks true associations. Also adjustment for social background factors could in fact be over-adjustment, since some of their effects on later mental health could be mediated by parental academic involvement.

## Conclusions

Parental involvement in their offspring’s studies may buffer against internalised mental health symptoms in adolescence, which may track into adulthood, at least among students with above average school grades. This has potentially large public health relevance since it indicates that interventions that strengthen parental involvement could decrease the population burden of mental health problems.

## Additional file

Additional file 1: Table S1. Model fit information for latent class growth analysis with different number of classes. **Figure S1.** Estimated mean trajectories for the models with three, four, five and six classes.

## References

[1] Whiteford HA, Degenhardt L, Rehm J, Baxter AJ, Ferrari AJ, Erskine HE, Charlson FJ, Norman RE, Flaxman AD, Johns N (2013). Global burden of disease attributable to mental and substance use disorders: findings from the Global Burden of Disease Study 2010. Lancet.

[2] Ferrari AJ, Charlson FJ, Norman RE, Patten SB, Freedman G, Murray CJ, Vos T, Whiteford HA (2013). Burden of depressive disorders by country, sex, age, and year: findings from the global burden of disease study 2010. PLoS Med.

[3] Sverige (2013). Socialstyrelsen, Statens folkhälsoinstitut: Folkhälsan i Sverige: årsrapport 2013 [Health in Sweden: The National Public Health Report 2013].

[4] West P, Sweeting H (2003). Fifteen, female and stressed: changing patterns of psychological distress over time. J Child Psychol Psychiatry.

[5] Birmaher B, Ryan ND, Williamson DE, Brent DA, Kaufman J (1996). Childhood and adolescent depression: a review of the past 10 years. Part II. J Am Acad Child Adolesc Psychiatry.

[6] Birmaher B, Ryan ND, Williamson DE, Brent DA, Kaufman J, Dahl RE, Perel J, Nelson B (1996). Childhood and adolescent depression: a review of the past 10 years. Part I. J Am Acad Child Adolesc Psychiatry.

[7] Jablonska B, Lindblad F, Ostberg V, Lindberg L, Rasmussen F, Hjern A (2012). A national cohort study of parental socioeconomic status and non-fatal suicidal behaviour – the mediating role of school performance. BMC Public Health.

[8] Alaraisanen A, Miettunen J, Lauronen E, Rasanen P, Isohanni M (2006). Good school performance is a risk factor of suicide in psychoses: a 35-year follow up of the Northern Finland 1966 Birth Cohort. Acta Psychiatr Scand.

[9] Bjorkenstam C, Weitoft GR, Hjern A, Nordstrom P, Hallqvist J, Ljung R (2011). School grades, parental education and suicide–a national register-based cohort study. J Epidemiol Community Health.

[10] Kosidou K, Dalman C, Fredlund P, Lee BK, Galanti R, Isacsson G, et al. School performance and the risk of suicide attempts in young adults: a longitudinal population-based study. Psychol Med. 2013;1–9. 10.1017/S0033291713001852 23883735

[11] Almquist YB (2013). School performance as a precursor of adult health: exploring associations to disease-specific hospital care and their possible explanations. Scand J Public Health.

[12] Ahren-Moonga J, Silverwood R, Klinteberg BA, Koupil I (2009). Association of higher parental and grandparental education and higher school grades with risk of hospitalization for eating disorders in females: the Uppsala birth cohort multigenerational study. Am J Epidemiol.

[13] Jonsson U, Goodman A, von Knorring AL, von Knorring L, Koupil I (2012). School performance and hospital admission due to unipolar depression: a three-generational study of social causation and social selection. Soc Psychiatry Psychiatr Epidemiol.

[14] Lehtinen H, Raikkonen K, Heinonen K, Raitakari OT, Keltikangas Jarvinen L (2006). School performance in childhood and adolescence as a predictor of depressive symptoms in adulthood. Sch Psychol Int.

[15] Fan XT, Chen M (2001). Parental involvement and students’ academic achievement: a meta-analysis. Educ Psychol Rev.

[16] Hill NE, Tyson DF (2009). Parental involvement in middle school: a meta-analytic assessment of the strategies that promote achievement. Dev Psychol.

[17] Wilder S (2014). Effects of parental involvement on academic achievement: a meta-synthesis. Educ Rev.

[18] Flouri E (2006). Parental interest in children's education, children's self-esteem and locus of control, and later educational attainment: twenty-six year follow-up of the 1970 British Birth Cohort. Brit J Educ Psychol.

[19] Bakoula C, Kolaitis G, Veltsista A, Gika A, Chrousos GP (2009). Parental stress affects the emotions and behaviour of children up to adolescence: a Greek prospective, longitudinal study. Stress.

[20] Mensah FK, Hobcraft J (2008). Childhood deprivation, health and development: associations with adult health in the 1958 and 1970 British prospective birth cohort studies. J Epidemiol Community Health.

[21] Hertzman C, Power C, Matthews S, Manor O (2001). Using an interactive framework of society and lifecourse to explain self-rated health in early adulthood. Soc Sci Med.

[22] Thomas C, Hypponen E, Power C (2008). Obesity and type 2 diabetes risk in midadult life: the role of childhood adversity. Pediatrics.

[23] Westerlund H, Gustafsson PE, Theorell T, Janlert U, Hammarstrom A (2013). Parental academic involvement in adolescence, academic achievement over the life course and allostatic load in middle age: a prospective population-based cohort study. J Epidemiol Community Health.

[24] Hammarstrom A, Janlert U (2012). Cohort profile: the Northern Swedish Cohort. Int J Epidemiol.

[25] Thorslund M, Warneryd B (1985). Methodological research in the Swedish surveys of living-conditions–problems of measurement and data-collection. Soc Indic Res.

[26] Johansson S (1970). The adult population’s state of health [in Swedish].

[27] Winefield HR, Hammarström A, Nygren K, Hägglöf B (2013). Internalized symptoms in adolescence as predictors of mental health in adulthood in the Northern Swedish cohort. Health.

[28] Jung T, Wickrama KAS (2008). An Introduction to latent class growth analysis and growth mixture modeling. Soc Personal Psychol Compass.

[29] Nagin DS (2005). Group-based modeling of development.

[30] Wickrama KA, Conger RD, Abraham WT (2008). Early family adversity, youth depressive symptom trajectories, and young adult socioeconomic attainment: a latent trajectory class analysis. Adv Life Course Res.

[31] Nylund KL, Asparouhov T, Muthén BO (2007). Deciding on the number of classes in latent class analysis and growth mixture modeling: a Monte Carlo simulation study. Struct Equ Model.

[32] Jokela M, Batty GD, Deary IJ, Gale CR, Kivimaki M (2009). Low childhood IQ and early adult mortality: the role of explanatory factors in the 1958 British Birth Cohort. Pediatrics.

