# Supplementary material for: Parental academic involvement in adolescence as predictor of mental health trajectories over the life course: a prospective population-based cohort study
Source: BMC Public Health. 2015 Jul 14;15:653. doi: 10.1186/s12889-015-1977-x (PMC4499905; doi:10.1186/s12889-015-1977-x)
Supplement: Additional file 1: Table S1. — Model fit information for latent class growth analysis with different number of classes. Figure S1. Estimated mean trajectories for the models with three, four, five and six classes. [file 12889_2015_1977_MOESM1_ESM.doc]

**Additional file 1**

**Table S1. Model fit information for latent class** growth analysis with different number of classes

| **Nr of**  **Classes** | **Loglikelihood** | **Akaike Information Criteria** | **Bayesian Information Criteria** | **LMR test*: value, p-value** | **Entropy** | **Group sizes** in per cent (average posterior probabilities)** |
| --- | --- | --- | --- | --- | --- | --- |
| **2** | -8605.871 | 17235.743 | 17295.107 | 612.858  0.0000 | 0.858 | 85.3 (0.97)  14.7 (0.90) |
| **3** | -8467.217 | 16966.433 | 17045.585 | 267.677  0.0063 | 0.948 | 85.6 (0.99)  10.2 (0.94)  4.2 (0.95) |
| **4** | -8348.724 | 16737.447 | 16836.387 | 228.754  0.0003 | 0.876 | 71.3 (0.94)  15.7 (0.84)  9.1 (0.97)  3.9 (0.97) |
| **5** | -8190.843 | 16429.687 | 16548.414 | 297.627  0.0002 | 0.943 | 57.6 (0.97)  26.2 (0.99)  7.9 (0.88)  5.4 (1.00)  3.0 (1.00) |
| **6** | -8152.395 | 16360.790 | 16499.305 | 74.226  0.0491 | 0.959 | 58.0 (0.97)  25.6 (0.99)  8.0 (0.93)  3.8 (0.97)  3.0 (1.00)  1.6 (0.92) |

* Lo-Mendell-Rubin adjusted likelihood ratio test

** based on most likely latent class membership

**Figure S1. Estimated mean trajectories for the models with three, four, five and six classes**


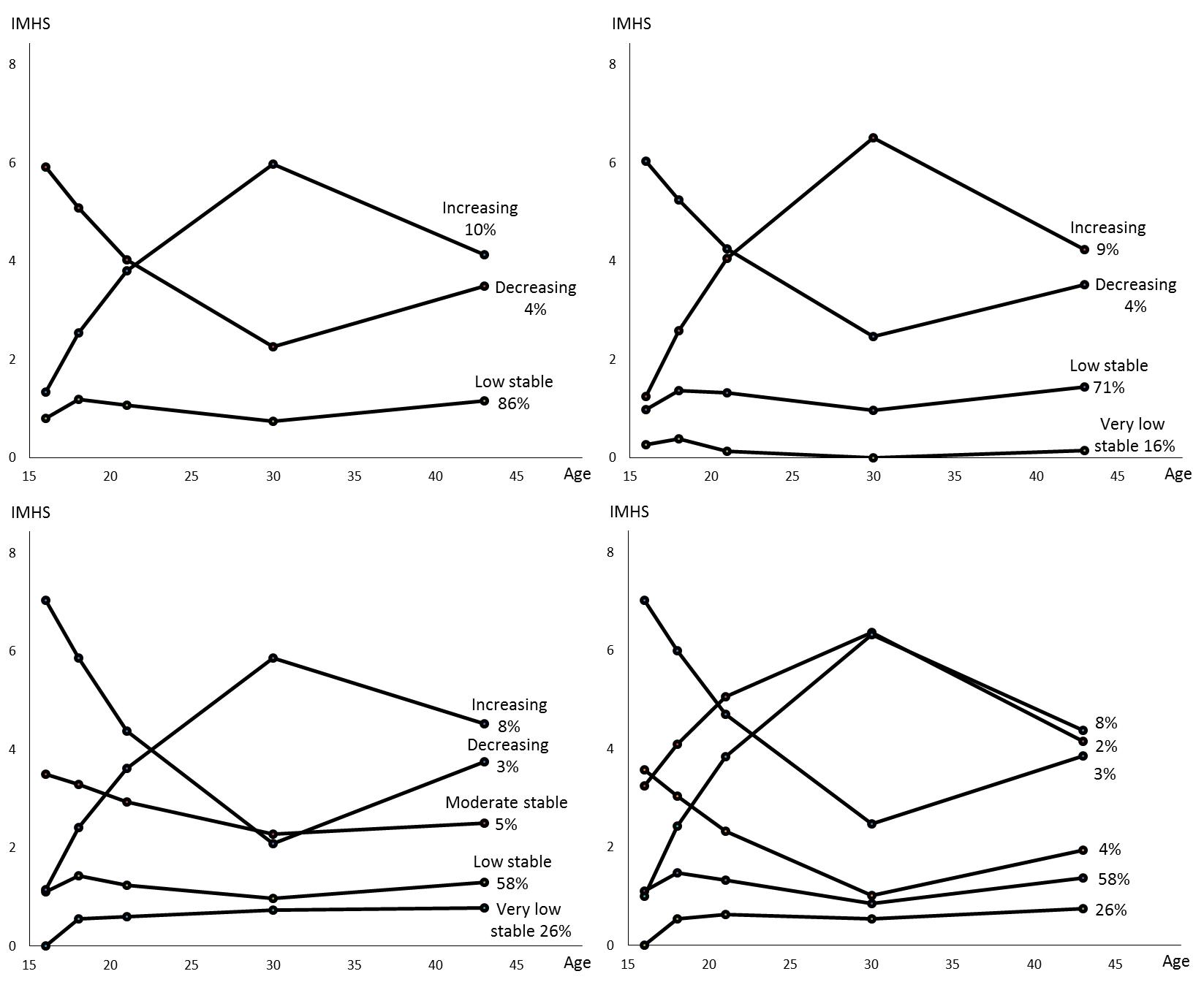


N.B. Percentages indicate the proportion of respondents who were classified into the different trajectories based on all participants in the study who could be classified into a trajectory, including those who were not part of the analytic sample in the present paper due to missing explanatory factors or covariates.
